# Health policy for sickle cell disease in Africa: experience from Tanzania on interventions to reduce under-five mortality

**DOI:** 10.1111/tmi.12428

**Published:** 2014-11-17

**Authors:** Julie Makani, Deogratias Soka, Stella Rwezaula, Marlene Krag, Janneth Mghamba, Kaushik Ramaiya, Sharon E. Cox, Scott D. Grosse

**Affiliations:** 1 Muhimbili Wellcome Programme, Muhimbili University of Health and Allied Sciences, Dar-es-Salaam, Tanzania; 2 Muhimbili National Hospital, Dar-es-Salaam, Tanzania; 3 University of Oxford, Oxford, UK; 4 Institute of International Health, Immunology and Microbiology, University of Copenhagen, Copenhagen, Denmark; 5 Ministry of Health and Social Welfare, Dar-es-Salaam, Tanzania; 6 London School of Hygiene and Tropical Medicine, London, UK; 7 Division of Blood Disorders, National Center on Birth Defects and Developmental Disabilities, Centers for Disease Control and Prevention, Atlanta, GA, USA

**Keywords:** public health, sickle cell anaemia, Africa, mortality, morbidity, trends, health policy, childhood mortality, newborn screening, pneumococcal vaccines

## Abstract

Tanzania has made considerable progress towards reducing childhood mortality, achieving a 57% decrease between 1980 and 2011. This epidemiological transition will cause a reduction in the contribution of infectious diseases to childhood mortality and increase in contribution from noncommunicable diseases (NCDs). Haemoglobinopathies are amongst the most common childhood NCDs, with sickle cell disease (SCD) being the commonest haemoglobinopathy in Africa. In Tanzania, 10 313 children with SCD under 5 years of age (U5) are estimated to die every year, contributing an estimated 7% of overall deaths in U5 children. Key policies that governments in Africa are able to implement would reduce mortality in SCD, focusing on newborn screening and comprehensive SCD care programmes. Such programmes would ensure that interventions such as prevention of infections using penicillin plus prompt diagnosis and treatment of complications are provided to all individuals with SCD.

## Introduction

Tanzania is one of 20 high-mortality countries that has achieved a 57% reduction in its under-five mortality rate (U5MR) from 158 per 1000 live births in 1990 to 68 in 2011 (Hill *et al.* 2012). Although most efforts targeted infectious causes of death, WHO projects that when U5MR falls to below 50 per thousand live births, countries will need to develop policies to directly reduce mortality due to non-communicable diseases (NCDs) (World Health Organization 2006a). Haemoglobinopathies are amongst the most common childhood NCDs globally. 75% of the 300 000 children born annually with sickle cell disease (SCD), one of the most severe haemoglobinopathies, are in Africa (Piel *et al.* 2013a). In this paper, we review the burden of SCD in Tanzania and discuss health policies to reduce childhood mortality in SCD.

### The burden of SCD in Tanzania

A working report published by WHO and the March of Dimes advised low and middle income countries (LMIC) to develop health programmes for haemoglobinopathies (World Health Organization 2006b), with the determination of the burden of SCD a key step to justify targeting limited resources towards evidence-based and appropriate policies.

The most accurate way to determine the birth prevalence of SCD is to conduct newborn screening (NBS). Within sub-Saharan Africa (SSA), no country has established a national universal NBS programme and therefore estimates rely on mathematical modelling using assumptions based on the frequency of the sickle gene. A recent report using this approach estimated the SCD birth prevalence in Tanzania to be 6 [interquartile range (IQR) 1–13] per 1000 births, equivalent to 11 877 (IQR: 9593-14 529) SCD births a year (Piel *et al.* 2013b). This ranks Tanzania as the fourth country in the world after Nigeria, Democratic Republic of Congo (DRC) and India, with the highest number of SCD births (Piel *et al.* 2013b). Like many African countries, the population prevalence of SCD in Tanzania is not known. However, Modell *et al.* suggested that if average survival for SCD reaches half the African norm, over six million Africans will be living with SCD (Modell & Darlison 2008). The life expectancy in Tanzania is 52 years, and half the African norm will be 21 years. A recent study in a tertiary hospital in Tanzania reported a median survival of 33 years in the SCD population seen at this hospital (Makani *et al.* 2011). This suggests that in some areas of Tanzania, there is increasing survival in SCD, which will result in a high population prevalence of SCD.

SCD is associated with high mortality, particularly in the U5 age group. Tanzania reported a SCD-specific mortality rate of 1.9 [95% confidence interval (CI): 1.5–2.9)] per hundred person-years of observation (100 PYO) (Makani *et al.* 2011). This compares to 0.15 per 100 PYO in the United Kingdom (UK) and 0.6 per 100 PYO in the USA (Telfer *et al.* 2007; Quinn *et al.* 2010). The highest SCD-specific mortality rates are in the U5 age group; 7.3 per 100 PYO in Tanzania compared to 0.72 and 0.43 per 100 PYO for children ages 0–2 and 2–4 in the USA (Quinn *et al.* 2004). Modell and Darlinson estimated that the contribution of haemoglobin disorders to U5 deaths is 3.4% worldwide and 6.4% in Africa (Modell & Darlison 2008). Based on these assumptions, 10 313 U5 individuals with SCD die annually in Tanzania.

## Health policies to reduce U5 mortality in SCD in Tanzania

With the reduction in U5MR in Tanzania, SCD has been recognised to be a disease of public health importance, requiring the introduction of interventions to reduce U5 mortality in SCD. Tanzania is developing a national policy for management of SCD (Ministry of Health & Social welfare Tanzania 2009). There is ongoing debate whether care of SCD should be integrated into existing health services or whether there should be disease-specific programmes (World Health Organization 2006a). WHO recommends that countries where the SCD birth prevalence exceeds 0.5 per 1000 births should develop separate SCD programmes. Although the SCD birth prevalence in Tanzania is 6 per 1000, Tanzania has strategically decided to integrate SCD into the NCD programme due to limitation of resources (Ministry of Health & Social welfare Tanzania 2009).

Comprehensive, dedicated SCD programmes that provide NBS, follow-up care, family and patient education and counselling, and prevention and treatment of complications can have a significant impact in reducing morbidity and mortality (Vichinsky 1991; Rahimy *et al.* 2003). Many African countries such as Benin, Ghana, Cameroon, DRC, Tanzania and Nigeria have established SCD centres (Rahimy *et al.* 2003; Akinyanju *et al.* 2005; Tshilolo *et al.* 2009; Makani *et al.* 2011). The experience of Jamaica indicates that even before interventions such as penicillin and pneumococcal vaccine became recognised or available, identification of SCD resulted in substantial decline in childhood mortality and increase in the number of individuals with SCD requiring care (Grosse *et al.* 2012). This is probably the result of parental and provider awareness and education (Grosse *et al.* 2012). Therefore, for countries with established SCD programmes, these sites will serve as national specialised treatment and referral centres and would support the development of SCD services at different healthcare levels within the country to ensure equitable access.

## Options for interventions

The third step is to identify appropriate interventions that could be implemented in Tanzania. Analyses of trends in mortality in SCD in the USA have shown dramatic reductions in childhood deaths (Yanni *et al.* 2009). The most dramatic reduction was up to 70% of deaths in the age group 0–3 years, most likely the result of identification of newborns with SCD by NBS and prevention of infection. Four options for SCD interventions are listed in Table 1 (Weatherall *et al.* 2006). Tanzania is working to implement interventions in Options 1 and 2; providing services for individuals diagnosed clinically with SCD, including penicillin prophylaxis and will be working to establish NBS and prevention of infection by promoting the use of penicillin and pneumococcal vaccination.

NBS for SCD could identify individuals with SCD at birth and subsequently enrol them into SCD comprehensive care programmes. The implementation plan for NBS for SCD that is being developed includes the establishment of capacity for laboratory diagnosis of SCD, with the proposal of integrating NBS with existing reproductive and child health programme. The use of dried blood spots for early infant diagnosis in HIV programmes has demonstrated that this approach is robust and can be effectively carried out in resource-limited settings. A pilot programme for the implementation of NBS for SCD is planned to start in 2015, with the aim of integrating the NBS policy into the reproductive and child health (RCH) programme.

The evidence of high burden of bacterial infections in SCD in Africa is unequivocal (Williams *et al.* 2009). Tanzania, like many African countries, has not introduced the use of prophylactic penicillin or pneumococcal vaccination amongst individuals with SCD. However, phenoxymethylpenicillin is cheap and is included in the National Essential Medicine List (NEMLIST). Therefore, the implementation of the use of phenoxymethylpenicillin in SCD children U5 years has started in Muhimbili National Hospital. With the inclusion of SCD in the national strategy for NCDs, the recommendation has been included in the training manual. In 2013, training of health workers all over the country started, which will ensure that the implementation of this recommendation reaches all levels of health care. With regards to prevention of infections by vaccination, in 2013, Tanzania included PCV in the expanded programme of immunization available to all children (http://www.afro.who.int/en/tanzania/press-materials/item/5205-tanzania-launches-the-introduction-of-two-new-vaccines-rotarix-and-pcv-13-with-a-call-to-ensure-all-children-are-vaccinated.html). Widespread use of PCV should lead to a reduction in the burden of U5 deaths from bacterial sepsis in children who receive PCV even without a diagnosis of SCD. Therefore, even without the introduction of NBS, the contribution of SCD deaths to the burden of U5 mortality will likely fall. Preventing infections and death from malaria is also an important component of preventing mortality in SCD, as malaria is associated with high mortality (Makani *et al.* 2010). Tanzania has made progress in protecting children from malaria, with the proportion of U5 children sleeping under a bed net increasing from 2% in 1999 to 64% in 2010.

## Conclusions

Of the four options of recommended interventional strategies, the introduction of NBS and prevention of infections using penicillin and pneumococcal vaccination are likely to be effective in reducing mortality in SCD in Tanzania. Tanzania and other African countries need to start planning appropriate health services and target interventions for SCD in the face of epidemiological transition.

## Figures and Tables

**Table 1:** Options for management of sickle cell disease ((Weatherall *et al*. 2006)

*Option one:* best possible patient care with the use of prophylactic penicillin following diagnosis, together with retrospective genetic counselling
*Option two:* best possible patient care, together with a neonatal screening programme and the use of penicillin for all homozygous babies, together with retrospective screening and genetic counselling
*Option three:* best possible patient care, together with a neonatal screening and the use of prophylactic penicillin from birth for homozygotes, together with population screening and prospective genetic counselling
*Option four:* as for option three, plus the availability of prenatal diagnosis, bone marrow transplantation, or both.
